# Renal mass biopsy using Raman spectroscopy identifies malignant and benign renal tumors: potential for pre-operative diagnosis

**DOI:** 10.18632/oncotarget.16419

**Published:** 2017-03-21

**Authors:** Yufei Liu, Zhebin Du, Jin Zhang, Haowen Jiang

**Affiliations:** ^1^ Department of Urology, Huashan Hospital, Fudan University, Shanghai, China; ^2^ Fudan Institute of Urology, Huashan Hospital, Fudan University, Shanghai, China; ^3^ Department of Urology, Ren Ji Hospital, School of Medicine, Shanghai Jiao Tong University, Shanghai, China

**Keywords:** Raman spectroscopy, renal tumor, biopsy, non-invasive, pre-operative diagnosis

## Abstract

The accuracy of renal mass biopsy to diagnose malignancy can be affected by multiple factors. Here, we investigated the feasibility of Raman spectroscopy to distinguish malignant and benign renal tumors using biopsy specimens. Samples were collected from 63 patients who received radical or partial nephrectomy, mass suspicious of cancer and distal parenchyma were obtained from resected kidney using an 18-gauge biopsy needle. Four Raman spectra were obtained for each sample, and Discriminant Analysis was applied for data analysis. A total of 383 Raman spectra were eventually gathered and each type of tumor had its characteristic spectrum. Raman could separate tumoral and normal tissues with an accuracy of 82.53%, and distinguish malignant and benign tumors with a sensitivity of 91.79% and specificity of 71.15%. It could classify low-grade and high-grade tumors with an accuracy of 86.98%. Besides, clear cell renal carcinoma was differentiated with oncocytoma and angiomyolipoma with accuracy of 100% and 89.25%, respectively. And histological subtypes of cell carcinoma were distinguished with an accuracy of 93.48%. When compared with final pathology and biopsy, Raman spectroscopy was able to correctly identify 7 of 11 “missed” biopsy diagnoses. These results suggested that Raman may serve as a promising non-invasive approach in the future for pre-operative diagnosis.

## INTRODUCTION

Increased use of imaging technique has led to frequent diagnosis of incidental renal masses, especially small ones. For patients who consider active surveillance or non-surgical treatments, renal mass biopsy (RMB) is often recommended to determine the pathological type of renal mass and guide treatment options. The accuracy of RMB depends on multiple factors, including interpretive skill of pathologists, the amount of specimens acquired, inherent sampling error, etc. According to previous studies, the accuracy could fluctuate from 79 to 100% [1–3], and non-diagnostic findings such as fibrosis or necrosis were found to be present in 15–22% cases [4–6]. Besides, RMB has limited accuracy when it comes to tumor grade classification- only 43 to 75% [7]. Thus, a technique that can provide objective evaluation of renal biopsy specimen is urgently needed.

Raman spectroscopy (RS) is a non-destructive optical technique, it relies on the inelastic scattering of photons derived from molecular bond vibrations [8]. When photons interact with molecular bonds, a part of them would change their frequencies to create a process called “Raman shift”. RS is able to reveal the chemical components and molecular structures of materials, and translate the information into a two-dimensional Raman spectrum. And it needs no sample pre-treatment or external labeling, and requires only a few seconds. Previous publications have presented the use of RS to detect the whole mass of renal tumor [9, 10], but no study on Raman's investigation of biopsy specimen was ever reported. In this context, we mimicked pre-operative RMB by collecting biopsy specimen from surgical removed kidney to determine whether RS is able to distinguish malignant and benign renal tumors, and serve as a complementary diagnostic tool in the future.

## RESULTS

Clinical and pathological statistics of the patients were displayed in Table 1. The final pathology included 42 clear cell renal cell carcinomas (RCC), 4 papillary RCC, 4 chromophobe RCC, 7 angiomyolipoma (AML) and 6 oncocytoma (RO). We obtained a total of 388 Raman spectra, 5 spectra were excluded from analysis due to strong fluorescent influence. Thus 383 spectra were eventually sent to Discriminant analysis (DA) (136 normal tissue, 165 clear cell RCC, 14 chromophobe RCC, 16 papillary RCC, 28 AML, 24 RO).

**Table 1:** Clinical and pathological statistics of patients

Variable	Value
No. of specimens	63
Patient gender:	
Male	41
Female	22
Patient age, mean, (yr)	64 (38–82)
Tumor size, mean, (cm)	5.2 (2–12)
Pathology:	
Benign, No	13
Angiomyolipoma	7
Oncocytoma	6
Malignant, No	50
Clear-cell	42
Papillary	4
Chromophobe	4
Tumor stage, No.	
pT1	35
pT2	15
pT3	0
Nodal invasion, No.	
N0	48
N1	2
Distant metastases, No.	
M0	50
M1	0
Tumor grade, No.	
1–2	45
3–4	5

**Figure 1:**
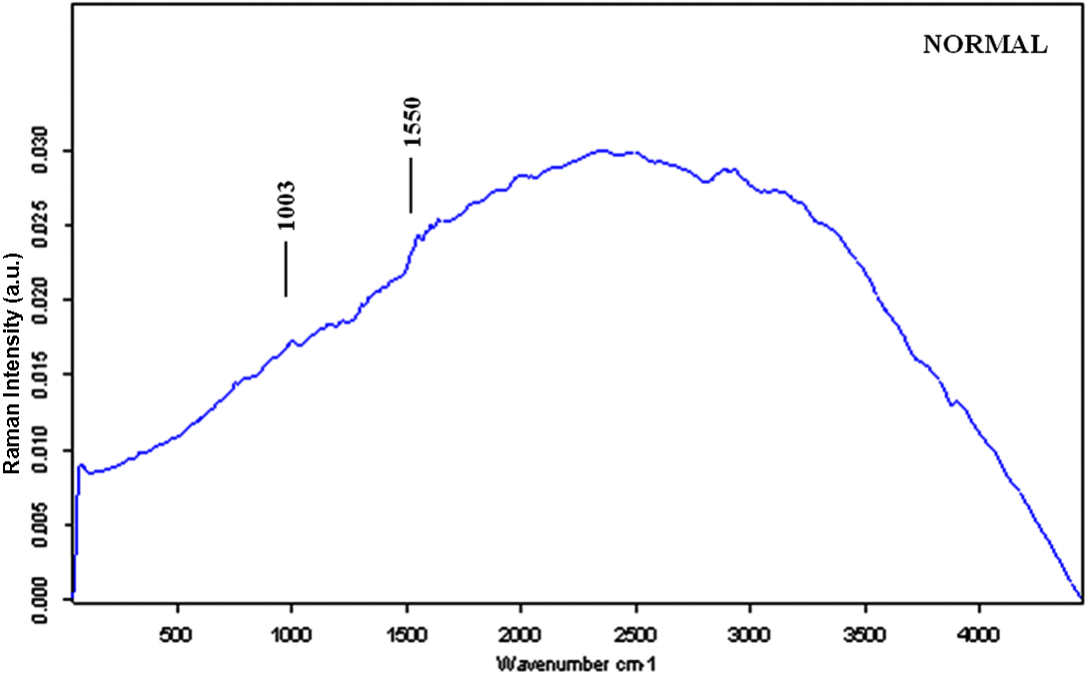
The mean Raman spectra of normal renal tissue

**Figure 2:**
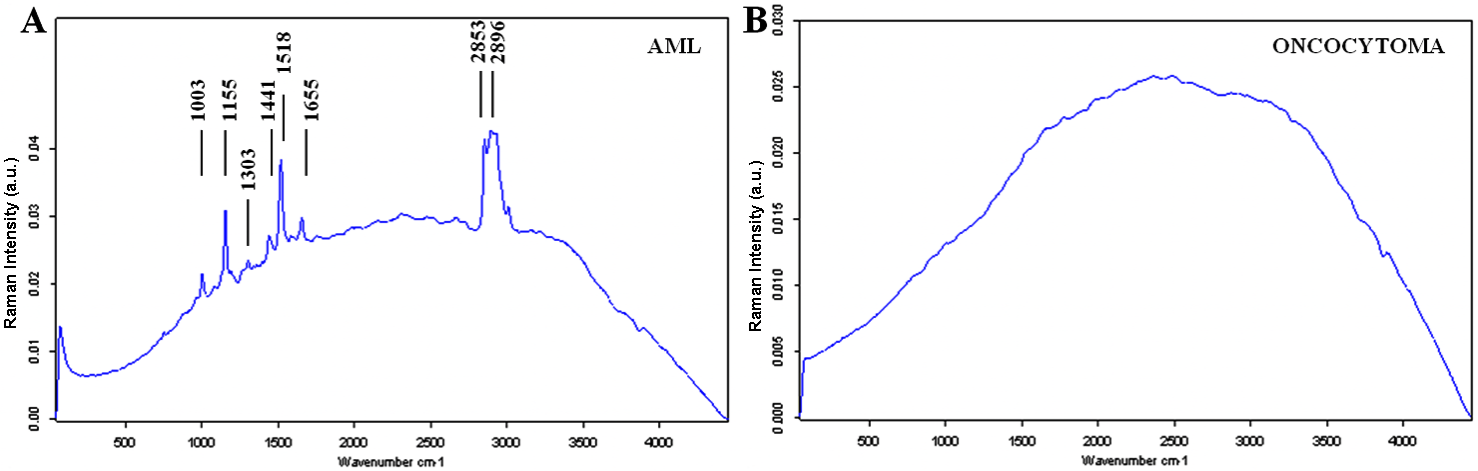
The mean Raman spectra of benign renal tumors (**A**) AML; (**B**) Oncocytoma.

**Figure 3:**
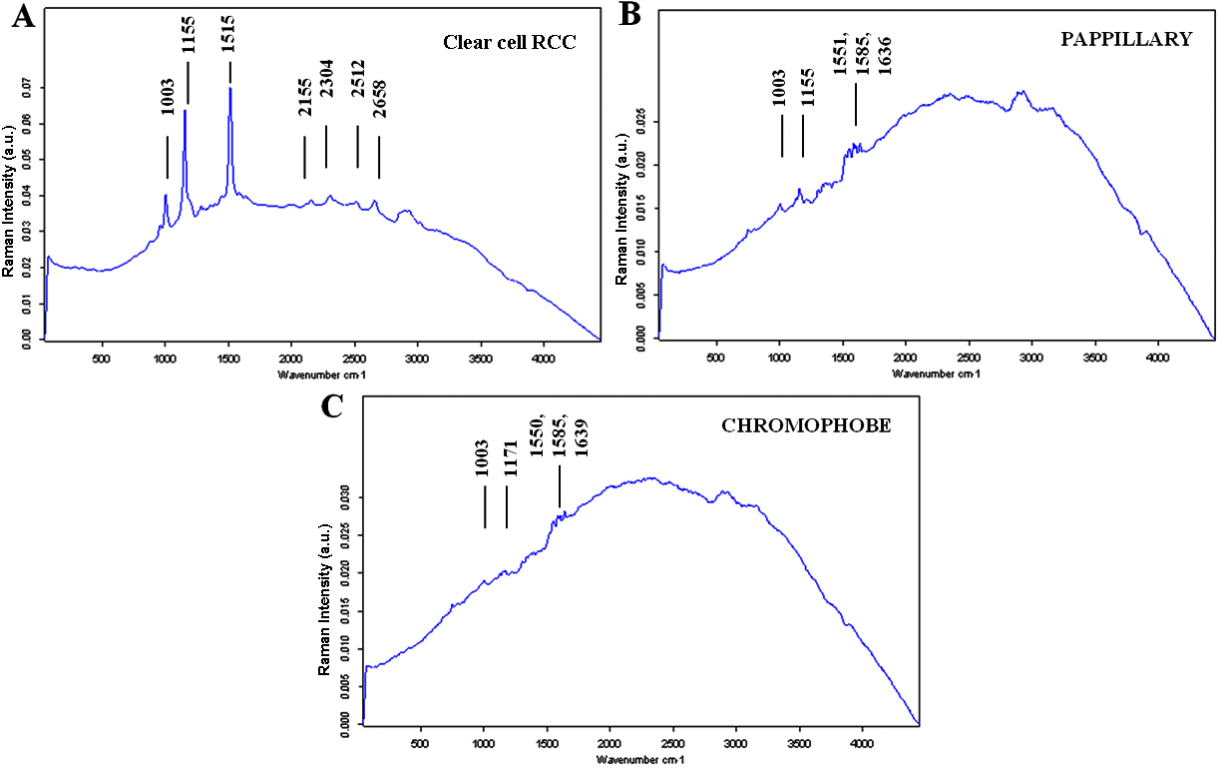
The mean Raman spectra of malignant renal tumors (**A**) Clear cell RCC; (**B**) Papillary RCC; (**C**) Chromophobe.

Characteristic Raman spectra were obtained for different types of renal tissues (Figures 1, 2, 3). The normal renal tissue presented a smooth wave with weak peaks were identified at 1,003 and 1,550 cm^−1^. RO, papillary RCC and chromophobe had similar spectral profile, but discrepancies existed: RO had no obvious peaks; chromophobe had small peaks at 1,003 and 1,170 cm^−1^, and three adjacent peaks at 1,500, 1,585, 1,639 cm^−1^; papillary RCC had similar peaks at 1,003, 1,500, 1,585 and 1,636 cm^−1^ (not 1,639 cm^−1^ as that of chromophobe), but a different peak at 1,155 cm^−1^ was identified. Clear cell RCC and AML both had significantly intensified peaks around 1,003, 1,155 and 1,515 cm^−1^ (1,518 cm^−1^ for AML), but clear cell RCC had 4 typical peaks at 2,155, 2,304, 2,512 and 2,658 cm^−1^, while AML had peaks at 1,303, 1,441, 1,665, 2,853 cm^−1^ and a flat peak around 2,896 cm^−1^. The assignments of Raman peaks were displayed in Table 2 [8, 11–15].

**Table 2:** Raman frequencies and their assignments

Peak position (cm^−1^)	Assignments
1003	Protein: Phenylalanine ring breath
1155~	Protein: C-C; C-N Stretching
1171~	Protein: C-H bend
1303~	Lipid, protein, nucleic acid
1441	Lipid: C-H_2_ deformation
1515	Protein: ß-Carotene C-C Stretching
1550,1551~	Protein: C = C; CN Stretching
1585	Phenylalanine C = C olefinic Stretching
1636	Fatty acid
1639~	Fatty acid
1665	Protein: Amide I
2155	/
2304	/
2512	/
2658	/
2854~	Lipid
2896	Lipid

Peaks at 2155, 2304, 2512 and 2658 cm^−1^ were undefined according to previous studies. One potential explanation would be the “fundamental, combination and overtone mode” [15]. Assuming 1003, 1155 and 1515 cm^−1^ of clear cell RCC were set as fundamentals U_1_, U_2_, U_3_, then 2155 ≈ 1003 + 1155 (combination U_1_ + U_2_), 2304 ≈ 2 × 1155 (overtone 2U_2_), 2512 ≈ 1003 + 1515 (combination U_1_ + U_3_), 2658 ≈ 1155 + 1515 (combination U_2_ + U_3_).

DA could identify tumoral and normal renal tissues with an accuracy of 82.53% (sensitivity of 87.85% and specificity of 77.21%): malignant tumors could be distinguished with normal tissues with an accuracy of 84.37% (sensitivity of 81.62% and specificity of 87.11%), and benign tumors could be distinguished with normal tissues with an accuracy of 89.59% (sensitivity of 92.65% and specificity of 86.54%). DA could separate malignant and benign tumors with an accuracy of 81.47% (sensitivity of 91.79% and specificity of 71.15%).

Clear cell RCC as the most common malignant renal tumor could be discriminated with normal tissues with an accuracy of 92.87% (sensitivity of 94.55% and specificity of 91.18%), and be separated with RO and AML with accuracy of 100% (sensitivity/specificity of 100%) and 89.25% (sensitivity of 96.36% and specificity of 82.14%), respectively.

Histological subtypes of RCC (defined as clear cell vs. papillary, chromophobe) were distinguished with an accuracy of 93.48% (sensitivity of 96.95% and specificity of 90.00%).

Low-grade and high-grade tumors could be distinguished with an accuracy of 86.98% (sensitivity of 96.19% and specificity of 77.78%).

Finally, among the 11 cases of missed diagnosis by biopsy, Raman was able to correctly identify 7 of them (Table 3).

**Table 3:** Raman's judgment on the “missed” biopsy diagnosis

	Final pathology	Biopsy Diagnosis	Raman
1	Clear cell RCC	Fibrosis	Clear cell RCC
2	Clear cell RCC	Fibrovascular tissue	/
3	Chromophobe	Fibrosis	/
4	Clear cell RCC	Degenerated tissue	/
5	Clear cell RCC	Degenerated tissue	Clear cell RCC
6	Clear cell RCC	Fibrovascular tissue	Clear cell RCC
7	Clear cell RCC	Degenerated tissue	Clear cell RCC
8	Clear cell RCC	Fibrosis	Clear cell RCC
9	Clear cell RCC	Blood Coagulum	/
10	Clear cell RCC	Fibrosis	Clear cell RCC
11	Clear cell RCC	Fibrovascular tissue	Clear cell RCC

Among the 11 cases of missed diagnosis, fibrosis, fibrovascular and degenerated tissue were mostly reported. Raman was able to correctly identify 7 of them.

## DISCUSSION

RS was initially applied in chemistry and gradually evolved to a scientific tool for investigating pathological tissues, it has advantage of providing rapid, non-invasive and even non-contact detection. The first use of RS in urology was documented in 1995 when Feld and his colleagues found that bladder cancer has denser nucleic acid and lower lipid content than normal bladder urothelium [16]. By far, RS has been utilized in detecting renal and prostatic cancers, urinary calculi and malignant cells [17–20]. In the current study, we demonstrated the availability of RS to distinguish different pathological types of renal tumors using biopsy specimens. The results were highlighted by Raman's ability to discriminate clear cell RCC and RO/AML. RO is the most common benign renal neoplasm which accounts for 3%–7% of all renal lesions [21], its differential diagnosis from clear cell RCC is sometimes hard because their imaging features can overlap on computed tomography [22, 23]. But our study proved that clear cell RCC and RO can be clearly separated by RS. AML was found to have typical peaks at 1,303, 1,441, 2,854 and 2,896 cm^−1^ corresponding to lipids [8], it complies with the fact that AML has abundant amounts of fat. Besides, it was promising to find that Raman correctly identified 7 of 11 missed diagnoses by biopsy, the reason may have to do with Raman's high special resolution and sensitivity which enable it to draw information from very tiny amount of tissues, which could hardly be realized by biopsy.

Another advantage of RS resides in its ability to provide chemical components and molecular structures of cells and tissues. Compared to previous studies [9, 10], we first provided characteristic Raman spectrum for each type of renal tumor. These spectra could potentially serve as chemical “fingerprints” and be used for pre-operative diagnosis. Besides, study of materials corresponding to these Raman peaks may provide new insights to the mechanisms of renal tumors.

However, several design limitations existed in our study. First, we collected specimen from surgical removed kidney, this was mainly due to limited cases of RMB performed, and pathological analysis takes priority over laboratory study. Secondly, we used distal renal parenchyma as control group, the reason had to do with the difficulty of getting approval from Institutional Review Board for using normal men's tissue. Thirdly, the study had a limited population, especially papillary and chromophobe RCC, and we did not further separate fat-poor AML. The population should be expanded in the future to ensure that the findings are reproducible.

This study was performed *ex vivo*. We are also working on Raman endoscopy system that connects Raman spectroscopy with a probe via optical fiber cable. This probe can pass down the instrument channel of endoscope and allows for real-time intra-operative analysis. We could use it for both pathological diagnosis and resection margin determination. But, the safety of RS still needs strict investigation before final clinical use. Though Raman is deemed as a non-invasive detective method, further studies must be done to ensure that Raman scanning will not cause any DNA damage to normal renal tissues.

In conclusion, this study was the first to evaluate renal biopsy specimen using Raman spectroscopy, and provided characteristic Raman spectrum for each type of tumor. Raman is able to distinguish malignant and benign tumors at a high accuracy without adding external labeling, and the spectra have potential to be used as “biomarkers” for pre-operative analysis. Raman may become a novel diagnostic approach and complements to RMB in the future to improve diagnostic accuracy.

## MATERIALS AND METHODS

### Specimens

This study was approved by Institutional Review Board (IRB) of Huashan Hospital, Fudan University. All patients were informed of the aim of this study, and signed the consent form. Between March and July 2015, 63 renal operations were performed in our urological center, including 34 radical nephrectomies and 29 partial nephrectomies. The surgeries were completed with an open access in 12 patients and laparoscopically in 51 patients. After removal of kidneys, renal masses suspicious of cancers were collected using an 18-gauge biopsy needle (GALLINI S.R.L., Italy), distal renal parenchymas from radical nephrectomies were collected as control group. The samples were stored at −80°C until RS analysis.

### Raman instrumentation

**Figure 4:**
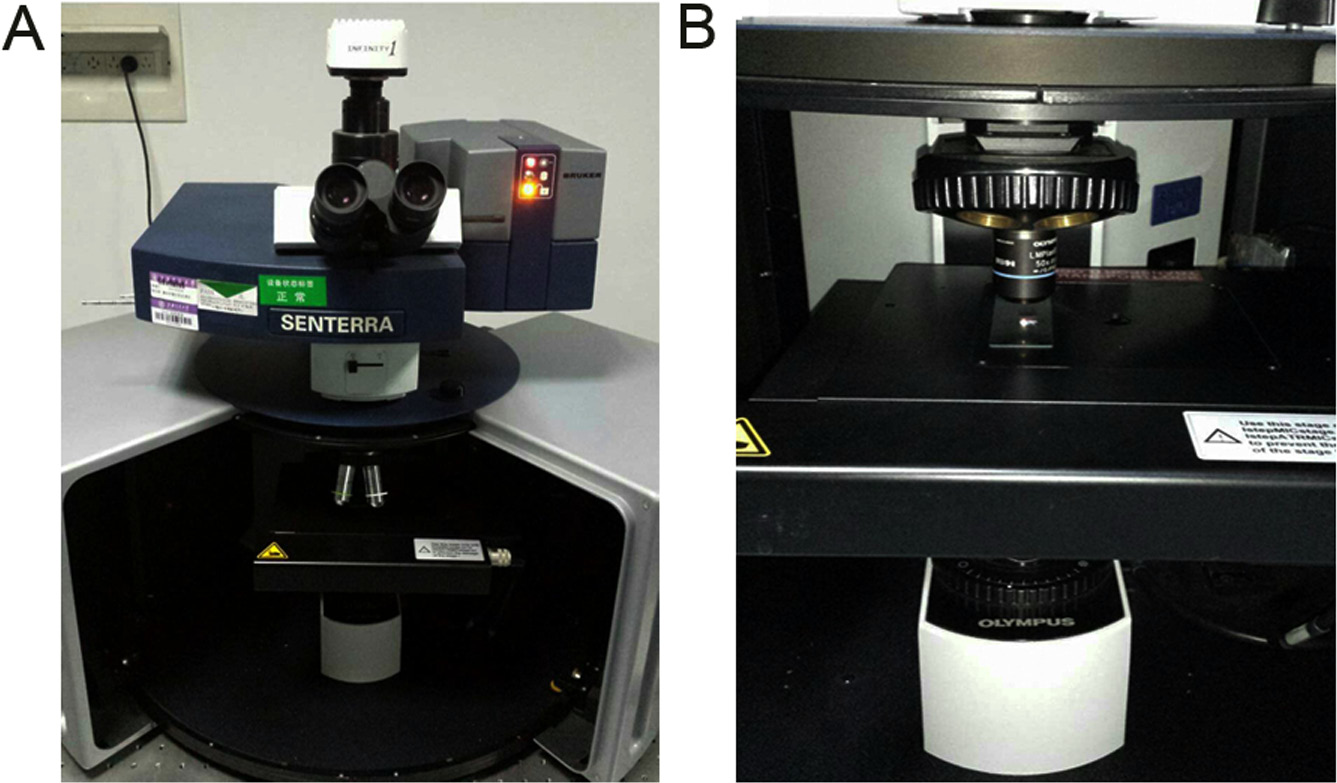
Actual setup of Raman spectroscopy (**A**) Overview of the Raman system; (**B**) Under working condition, the biopsy was placed on the sample stage with the laser focused onto it.

Raman spectroscopy (Senterra, Bruker Optics) was used to interrogate renal tissues (Figure 4), the system was described before [24]. Briefly, it was comprised of two components: (1) a spectrometer that equipped with lasers of three different excitation wavelengths and a thermoelectrically cooled, charge-coupled device (CCD) detector; (2) a microscope (model-BX51, Olympus) that equipped with a digital video camera capable of visualizing samples on a motorized sample stage. Under working condition, the laser was coupled into the microscope via an optical fiber and focused onto the samples. Then, the Raman signal was captured by the CCD detector, translated into Raman spectra and displayed on a personal computer.

### Raman measurements

Specimens were brought to Raman analysis at room temperature. Raman spectra were acquired by focusing the laser onto the surface of the renal tissues. Four spectra were collected per specimen from different locations. The laser used in this study was 532 nm in wavelength and 10 mW in power, and the acquisition period of each spectrum was 10s, with a 4 cm^−1^ spectral resolution over a 45–4450 cm^−1^ Raman shift range. After analysis, the biopsy samples were sent to pathological diagnosis, and the results were given by two independent skilled uropathologists at our hospital.

OPUS software 7.2 was used to record Raman spectra. To ensure all Raman spectra were comparable, several data processing steps were performed before analysis: (1) spectral calibration using the known spectra of silicon slice; (2) correction of spectral response of system using a tungsten white light source diffusely scattered by a reflectance standard BaSO_4_; (3) fluorescence background removal using a fifth order polynomial fitting; (4) baseline correction using a stretched rubber band between the spectrum endpoints that follows the spectrum minima; (5) data normalization by dividing each spectral point by the area of the total intensity of the spectrum. The Raman spectra were corresponded to the final surgical pathology.

### Statistical analysis

Our objective was to generate a diagnostic algorithm using RS capable of distinguishing (1) renal tumors and normal parenchyma; (2) malignant and benign tumors; (3) different pathological types of renal cell carcinoma (RCC); (4) high-grade (Fuhrman III–IV) and low-grade (Fuhrman I–II) tumors. For that purpose, Discriminant Analysis (DA) was used to generate this diagnostic algorithm, then a standard ‘leave-one spectrum out’ cross-validation was used to test the predictive capability of RS [25]. In that procedure, all spectra except one were used to test the DA and the remaining spectrum was left for continuing testing. The procedure was repeated with alternation of spectra. At the end, a cross-validation score that represented the capability of a single Raman spectrum to classify assessed biological sample was acquired. All analysis was performed in Matlab^®^ Software (Mathworks Inc., USA).
